# Supplementary material for: Mosquito abundance and diversity in central Ohio, USA vary among stormwater wetlands, retention ponds, and detention ponds and their associated environmental parameters
Source: PLoS One. 2024 Jun 25;19(6):e0305399. doi: 10.1371/journal.pone.0305399 (PMC11198753; doi:10.1371/journal.pone.0305399)
Supplement: S2 File — (HTML) [file pone.0305399.s002.html]

Mosquito abundance and diversity vary among stormwater control measures and associated environmental characteristics in central Ohio


# Mosquito abundance and diversity vary among stormwater control measures and associated environmental characteristics in central Ohio

#### James Radl, Luis Martinez Villegas, Joseph S. Smith, R. Andrew Tirpak, Kayla I. Perry, Deirdre Wetmore, Elena Tunis, Jack Smithburger, Henry Schuellerman, Dom Magistrado, Ryan J. Winston, Sarah M. Short

Load Data and edit classes of variables

Before beginning, download the S1 File and save the raw data as a .csv file

```
scmdata<-read.csv("D:/OneDrive - The Ohio State University/Radl-Shared/P5-StormwaterTreatment/Manuscript/Additional Files/S1 File.csv")
scmdata$Date<-as.Date(scmdata$Date)
scmdata$Period<-as.factor(scmdata$Period)
scmdata$Site<-as.factor(scmdata$Site)
scmdata$Location<-as.factor(scmdata$Location)
scmdata$SCM_Type<-as.factor(scmdata$SCM_Type)

#Creating color palettes for figures
type.colors<-c("#00A600", "#FF9900", "#9900FF")  
library(paletteer)
```

```
## Warning: package 'paletteer' was built under R version 4.1.3
```

```
hm.colors<-rev(paletteer_c("ggthemes::Orange-Blue-White Diverging", 1000))
```

Sum site totals and generate the mosquito “abundance per sample” variable (APS)

```
sitetotals<-aggregate(x=scmdata[,14:25], by=list(scmdata$Site), FUN=sum, na.rm=TRUE) #Sum species abundance for each site across all samples
names(sitetotals)[names(sitetotals) == "Group.1"]<-"Site" #renaming site column
sitetotals$SCM_Type<-as.factor(ifelse(substr(sitetotals$Site, 1, 1)=="P", "RP", 
                                      ifelse(substr(sitetotals$Site, 1, 1)=="D", "DP", "CW"))) #re-adding SCM_Type
print(sitetotals[,1:11])
```

```
##    Site Ae.ve Ps.fe Cx.te Cx.er Cx.pi Cx.re Cx.sa Cs.in An. Ur.sa
## 1  DP11    31     0    36     0   526     9     0     0  14     0
## 2  DP12     0     0     0     0     0     0     0     0  16     0
## 3  DP13     1     0     2     0     0     0     0     0  14     0
## 4  DP14     0     0     0     5     0     0     0     0  15     0
## 5  DP15     0     0     0     0     0     0     0     0   3     0
## 6  DP17     9     0     0     0    28    14     2     0   2     0
## 7  DP18     0     0     4    22     0     0     0     0  22     0
## 8  DP20   212     0     0     0   181     1     2     0   5     0
## 9   DP4     0     0     0     0    36     0     0     0   1     0
## 10  DP9     0     0     9     0     0     0     0     0   3     0
## 11  P12     0     0   369    18     2    95     0     0  35     4
## 12  P14     0     0     6   176     0     0     0     0  87    10
## 13  P15     0     0     0     5     0     0     0     0   0     0
## 14  P19     0     0     0     0     2     0     0     0   0     0
## 15   P2     0     0     6     0     0     0     0     0   5     1
## 16  P21     4     0     4    25     7     0     0     0  26     1
## 17  P27     0     0     2   196     0     0     0     0  79     0
## 18  P28     0     0     9   122     0     0     0     0  82     0
## 19   P5     0     0     0     0     0     0     0     0   0     0
## 20   P7     0     0    12   121     0     0     0     0  98     0
## 21   W1    79     0    54     7    16     0     8     0  36     3
## 22  W10     0     0    10    20     0     0     0     0  58     0
## 23  W11    12     0    16     0     1     0     0     0   7     6
## 24  W12   147     0    65    30     1     0     1     0  49    25
## 25  W13     5     0     8     9     0     0     0     0  34     0
## 26  W20    59     2   919     1   288   112   161    14  44    12
## 27   W4     0     0    34    12     2     0     0     0  34     0
## 28   W5     0     0     6    16     1     0     3     0  77     0
## 29   W6    23     0   232     8    37     0     3     0  12     4
## 30   W9     0     0    34     4     0     0     0     0  19     1
```

Create separate dataframe for each year

```
#2021 Data
scmdata21<-scmdata[scmdata$Date<"2022-01-01",]
sitetotals21<-aggregate(x=scmdata21[,14:25], by=list(scmdata21$Site), FUN=sum, na.rm=T)
names(sitetotals21)[names(sitetotals21) == "Group.1"]<-"Site"
sitetotals21$SCM_Type<-as.factor(ifelse(substr(sitetotals21$Site, 1, 1)=="P", "RP", 
                                      ifelse(substr(sitetotals21$Site, 1, 1)=="D", "DP", "CW")))
print(sitetotals21[,1:11])
```

```
##    Site Ae.ve Ps.fe Cx.te Cx.er Cx.pi Cx.re Cx.sa Cs.in An. Ur.sa
## 1  DP11     6     0    27     0   143     1     0     0   7     0
## 2  DP12     0     0     0     0     0     0     0     0   9     0
## 3  DP13     1     0     0     0     0     0     0     0   1     0
## 4  DP14     0     0     0     0     0     0     0     0   0     0
## 5  DP15     0     0     0     0     0     0     0     0   3     0
## 6  DP17     0     0     0     0     0     0     0     0   0     0
## 7  DP18     0     0     4    19     0     0     0     0  13     0
## 8  DP20   183     0     0     0    17     0     0     0   0     0
## 9   DP4     0     0     0     0     0     0     0     0   0     0
## 10  DP9     0     0     1     0     0     0     0     0   3     0
## 11  P12     0     0   349    10     0     0     0     0  23     4
## 12  P14     0     0     5    34     0     0     0     0  27     0
## 13  P15     0     0     0     5     0     0     0     0   0     0
## 14  P19     0     0     0     0     0     0     0     0   0     0
## 15   P2     0     0     0     0     0     0     0     0   2     0
## 16  P21     4     0     2     9     0     0     0     0   8     0
## 17  P27     0     0     1    27     0     0     0     0  46     0
## 18  P28     0     0     8    18     0     0     0     0  17     0
## 19   P5     0     0     0     0     0     0     0     0   0     0
## 20   P7     0     0     0    61     0     0     0     0  51     0
## 21   W1    79     0    52     3     0     0     0     0  10     2
## 22  W10     0     0    10     4     0     0     0     0  57     0
## 23  W11     4     0    16     0     0     0     0     0   5     6
## 24  W12   143     0    65    29     1     0     0     0  39    24
## 25  W13     5     0     7     3     0     0     0     0  18     0
## 26  W20     0     0   511     1   211    10    24     0  19    11
## 27   W4     0     0    15     6     1     0     0     0  15     0
## 28   W5     0     0     0     1     1     0     0     0  12     0
## 29   W6     0     0   181     1     5     0     0     0   7     3
## 30   W9     0     0    22     3     0     0     0     0  13     0
```

```
#2022 Data
scmdata22<-scmdata[scmdata$Date>"2022-01-01",]
sitetotals22<-aggregate(x=scmdata22[,14:25], by=list(scmdata22$Site), FUN=sum, na.rm=T)
names(sitetotals22)[names(sitetotals22) == "Group.1"]<-"Site"
sitetotals22$SCM_Type<-as.factor(ifelse(substr(sitetotals22$Site, 1, 1)=="P", "RP", 
                                      ifelse(substr(sitetotals22$Site, 1, 1)=="D", "DP", "CW")))
print(sitetotals22[,1:11])
```

```
##    Site Ae.ve Ps.fe Cx.te Cx.er Cx.pi Cx.re Cx.sa Cs.in An. Ur.sa
## 1  DP11    25     0     9     0   383     8     0     0   7     0
## 2  DP12     0     0     0     0     0     0     0     0   7     0
## 3  DP13     0     0     2     0     0     0     0     0  13     0
## 4  DP14     0     0     0     5     0     0     0     0  15     0
## 5  DP15     0     0     0     0     0     0     0     0   0     0
## 6  DP17     9     0     0     0    28    14     2     0   2     0
## 7  DP18     0     0     0     3     0     0     0     0   9     0
## 8  DP20    29     0     0     0   164     1     2     0   5     0
## 9   DP4     0     0     0     0    36     0     0     0   1     0
## 10  DP9     0     0     8     0     0     0     0     0   0     0
## 11  P12     0     0    20     8     2    95     0     0  12     0
## 12  P14     0     0     1   142     0     0     0     0  60    10
## 13  P15     0     0     0     0     0     0     0     0   0     0
## 14  P19     0     0     0     0     2     0     0     0   0     0
## 15   P2     0     0     6     0     0     0     0     0   3     1
## 16  P21     0     0     2    16     7     0     0     0  18     1
## 17  P27     0     0     1   169     0     0     0     0  33     0
## 18  P28     0     0     1   104     0     0     0     0  65     0
## 19   P5     0     0     0     0     0     0     0     0   0     0
## 20   P7     0     0    12    60     0     0     0     0  47     0
## 21   W1     0     0     2     4    16     0     8     0  26     1
## 22  W10     0     0     0    16     0     0     0     0   1     0
## 23  W11     8     0     0     0     1     0     0     0   2     0
## 24  W12     4     0     0     1     0     0     1     0  10     1
## 25  W13     0     0     1     6     0     0     0     0  16     0
## 26  W20    59     2   408     0    77   102   137    14  25     1
## 27   W4     0     0    19     6     1     0     0     0  19     0
## 28   W5     0     0     6    15     0     0     3     0  65     0
## 29   W6    23     0    51     7    32     0     3     0   5     1
## 30   W9     0     0    12     1     0     0     0     0   6     1
```

Compare mosquito APS with SCM type using Zero-Altered Generalized Linear Mixed Models Note that GLMMs with negative bionomial distributions do not accept decimals, so APS is multiplied and rounded to give an average count per hundred

```
library(glmmTMB)
library(car)
```

```
## Warning: package 'car' was built under R version 4.1.2
```

```
## Loading required package: carData
```

```
library(emmeans)
```

```
## Warning: package 'emmeans' was built under R version 4.1.3
```

```
library(ggplot2)

#2021
siteperiodaps21<-aggregate(scmdata21[,25], by=list(scmdata21$Period, scmdata21$Site), FUN=mean, na.rm=T) #Need to calculate APS (=mean of Total Mosquitoes) per Period and Site
names(siteperiodaps21)[names(siteperiodaps21) == "Group.1"]<-"Period"
names(siteperiodaps21)[names(siteperiodaps21) == "Group.2"]<-"Site"
siteperiodaps21$SCM_Type<-as.factor(ifelse(substr(siteperiodaps21$Site, 1, 1)=="P", "RP", 
                                      ifelse(substr(siteperiodaps21$Site, 1, 1)=="D", "DP", "CW")))
siteperiodaps21$aps100<-round(x=(100*siteperiodaps21$x), digits=0)
head(siteperiodaps21)
```

```
##   Period Site     x SCM_Type aps100
## 1      1 DP11  0.25       DP     25
## 2      2 DP11  4.50       DP    450
## 3      3 DP11  2.00       DP    200
## 4      4 DP11  6.25       DP    625
## 5      5 DP11 27.50       DP   2750
## 6      6 DP11  3.25       DP    325
```

```
glmtypevsaps21<-glmmTMB(aps100~SCM_Type+Period+(1|Site), data=siteperiodaps21, family=truncated_nbinom2, ziformula=~SCM_Type+Period+(1|Site)) #Generate the ZA-GLMM
summary(glmtypevsaps21)
```

```
##  Family: truncated_nbinom2  ( log )
## Formula:          aps100 ~ SCM_Type + Period + (1 | Site)
## Zero inflation:          ~SCM_Type + Period + (1 | Site)
## Data: siteperiodaps21
## 
##      AIC      BIC   logLik deviance df.resid 
##   1847.1   1934.9   -898.5   1797.1      223 
## 
## Random effects:
## 
## Conditional model:
##  Groups Name        Variance Std.Dev.
##  Site   (Intercept) 0.772    0.8786  
## Number of obs: 248, groups:  Site, 30
## 
## Zero-inflation model:
##  Groups Name        Variance Std.Dev.
##  Site   (Intercept) 3.048    1.746   
## Number of obs: 248, groups:  Site, 30
## 
## Dispersion parameter for truncated_nbinom2 family ():  1.1 
## 
## Conditional model:
##             Estimate Std. Error z value Pr(>|z|)    
## (Intercept)   4.2974     0.5486   7.833 4.76e-15 ***
## SCM_TypeDP    0.5491     0.5390   1.019   0.3083    
## SCM_TypeRP   -0.3158     0.4906  -0.644   0.5198    
## Period2       0.6281     0.5866   1.071   0.2842    
## Period3       0.8478     0.5607   1.512   0.1305    
## Period4       0.7060     0.5665   1.246   0.2126    
## Period5       1.1002     0.5622   1.957   0.0504 .  
## Period6       0.7691     0.5428   1.417   0.1565    
## Period7       1.0176     0.5692   1.788   0.0738 .  
## Period8       0.8293     0.5475   1.515   0.1298    
## Period9       0.4978     0.5609   0.888   0.3748    
## ---
## Signif. codes:  0 '***' 0.001 '**' 0.01 '*' 0.05 '.' 0.1 ' ' 1
## 
## Zero-inflation model:
##             Estimate Std. Error z value Pr(>|z|)    
## (Intercept)   0.3413     0.8413   0.406 0.684995    
## SCM_TypeDP    3.0218     0.9832   3.073 0.002118 ** 
## SCM_TypeRP    2.3660     0.9380   2.522 0.011655 *  
## Period2      -0.9698     0.7854  -1.235 0.216898    
## Period3      -1.7373     0.7853  -2.212 0.026941 *  
## Period4      -1.5035     0.7851  -1.915 0.055470 .  
## Period5      -2.0087     0.7916  -2.537 0.011169 *  
## Period6      -3.0351     0.8278  -3.667 0.000246 ***
## Period7      -2.9042     0.8646  -3.359 0.000782 ***
## Period8      -3.5008     0.8676  -4.035 5.46e-05 ***
## Period9      -2.4764     0.8018  -3.088 0.002012 ** 
## ---
## Signif. codes:  0 '***' 0.001 '**' 0.01 '*' 0.05 '.' 0.1 ' ' 1
```

```
Anova(glmtypevsaps21, component="cond") #non-significant for either period or type
```

```
## Analysis of Deviance Table (Type II Wald chisquare tests)
## 
## Response: aps100
##           Chisq Df Pr(>Chisq)
## SCM_Type 2.2427  2     0.3258
## Period   6.0370  8     0.6431
```

```
Anova(glmtypevsaps21, component="zi") #significant for period and type
```

```
## Analysis of Deviance Table (Type II Wald chisquare tests)
## 
## Response: aps100
##           Chisq Df Pr(>Chisq)   
## SCM_Type 10.559  2   0.005095 **
## Period   25.022  8   0.001542 **
## ---
## Signif. codes:  0 '***' 0.001 '**' 0.01 '*' 0.05 '.' 0.1 ' ' 1
```

```
emmeans(glmtypevsaps21, component="zi", pairwise~SCM_Type) #wetland is significantly different from both
```

```
## $emmeans
##  SCM_Type emmean    SE  df lower.CL upper.CL
##  CW       -1.674 0.670 223  -2.9936   -0.354
##  DP        1.348 0.695 223  -0.0218    2.718
##  RP        0.692 0.637 223  -0.5627    1.947
## 
## Results are averaged over the levels of: Period 
## Results are given on the logit (not the response) scale. 
## Confidence level used: 0.95 
## 
## $contrasts
##  contrast estimate    SE  df t.ratio p.value
##  CW - DP    -3.022 0.983 223  -3.073  0.0067
##  CW - RP    -2.366 0.938 223  -2.522  0.0330
##  DP - RP     0.656 0.933 223   0.703  0.7619
## 
## Results are averaged over the levels of: Period 
## Results are given on the log odds ratio (not the response) scale. 
## P value adjustment: tukey method for comparing a family of 3 estimates
```

```
emmeans(glmtypevsaps21, component="zi", pairwise~Period)
```

```
## $emmeans
##  Period emmean    SE  df lower.CL upper.CL
##  1       2.137 0.695 223   0.7675   3.5069
##  2       1.167 0.626 223  -0.0659   2.4007
##  3       0.400 0.603 223  -0.7891   1.5889
##  4       0.634 0.612 223  -0.5730   1.8403
##  5       0.129 0.606 223  -1.0663   1.3234
##  6      -0.898 0.624 223  -2.1270   0.3313
##  7      -0.767 0.678 223  -2.1029   0.5688
##  8      -1.364 0.661 223  -2.6665  -0.0606
##  9      -0.339 0.602 223  -1.5262   0.8478
## 
## Results are averaged over the levels of: SCM_Type 
## Results are given on the logit (not the response) scale. 
## Confidence level used: 0.95 
## 
## $contrasts
##  contrast          estimate    SE  df t.ratio p.value
##  Period1 - Period2    0.970 0.785 223   1.235  0.9479
##  Period1 - Period3    1.737 0.785 223   2.212  0.4013
##  Period1 - Period4    1.504 0.785 223   1.915  0.6040
##  Period1 - Period5    2.009 0.792 223   2.537  0.2200
##  Period1 - Period6    3.035 0.828 223   3.667  0.0092
##  Period1 - Period7    2.904 0.865 223   3.359  0.0253
##  Period1 - Period8    3.501 0.868 223   4.035  0.0024
##  Period1 - Period9    2.476 0.802 223   3.088  0.0567
##  Period2 - Period3    0.767 0.722 223   1.063  0.9788
##  Period2 - Period4    0.534 0.725 223   0.736  0.9982
##  Period2 - Period5    1.039 0.727 223   1.430  0.8853
##  Period2 - Period6    2.065 0.757 223   2.728  0.1441
##  Period2 - Period7    1.934 0.798 223   2.423  0.2767
##  Period2 - Period8    2.531 0.797 223   3.177  0.0440
##  Period2 - Period9    1.507 0.733 223   2.057  0.5058
##  Period3 - Period4   -0.234 0.710 223  -0.329  1.0000
##  Period3 - Period5    0.271 0.707 223   0.384  1.0000
##  Period3 - Period6    1.298 0.729 223   1.780  0.6953
##  Period3 - Period7    1.167 0.773 223   1.509  0.8504
##  Period3 - Period8    1.763 0.766 223   2.301  0.3458
##  Period3 - Period9    0.739 0.708 223   1.044  0.9810
##  Period4 - Period5    0.505 0.713 223   0.708  0.9986
##  Period4 - Period6    1.532 0.739 223   2.074  0.4939
##  Period4 - Period7    1.401 0.783 223   1.789  0.6894
##  Period4 - Period8    1.997 0.777 223   2.571  0.2048
##  Period4 - Period9    0.973 0.717 223   1.357  0.9124
##  Period5 - Period6    1.026 0.728 223   1.411  0.8928
##  Period5 - Period7    0.896 0.774 223   1.157  0.9644
##  Period5 - Period8    1.492 0.764 223   1.954  0.5771
##  Period5 - Period9    0.468 0.709 223   0.660  0.9992
##  Period6 - Period7   -0.131 0.783 223  -0.167  1.0000
##  Period6 - Period8    0.466 0.762 223   0.611  0.9995
##  Period6 - Period9   -0.559 0.721 223  -0.775  0.9974
##  Period7 - Period8    0.597 0.813 223   0.733  0.9983
##  Period7 - Period9   -0.428 0.767 223  -0.557  0.9998
##  Period8 - Period9   -1.024 0.754 223  -1.358  0.9120
## 
## Results are averaged over the levels of: SCM_Type 
## Results are given on the log odds ratio (not the response) scale. 
## P value adjustment: tukey method for comparing a family of 9 estimates
```

```
siteperiodaps21$pos<-ifelse(siteperiodaps21$aps100>0, siteperiodaps21$x, NA) #Adding column for positive only APS
siteperiodaps21$logpos<-log10(siteperiodaps21$pos) #Log transforming for visualizing
#Box plot of positive APS values for each Period and Site
ggplot() +
  geom_boxplot(data=siteperiodaps21, aes(x=Period, y=logpos, color=SCM_Type)) +
  theme_classic() +
  scale_color_manual("SCM Type", values=type.colors, labels=c("Const. Wetland", "Detention Pond", "Retention Pond")) + 
  xlab("Period") + ylab(expression(Log["10"]*APS)) + ylim(-1,2)
```

```
## Warning: Removed 138 rows containing non-finite values (`stat_boxplot()`).
```

```
#2022 Data
siteperiodaps22<-aggregate(scmdata22[,25], by=list(scmdata22$Period, scmdata22$Site), FUN=mean, na.rm=T)
names(siteperiodaps22)[names(siteperiodaps22) == "Group.1"]<-"Period"
names(siteperiodaps22)[names(siteperiodaps22) == "Group.2"]<-"Site"
siteperiodaps22$SCM_Type<-as.factor(ifelse(substr(siteperiodaps22$Site, 1, 1)=="P", "RP", 
                                      ifelse(substr(siteperiodaps22$Site, 1, 1)=="D", "DP", "CW")))
siteperiodaps22$aps100<-round(x=(100*siteperiodaps22$x), digits=0)

glmtypevsaps22<-glmmTMB(aps100~SCM_Type+Period+(1|Site), data=siteperiodaps22, family=truncated_nbinom2, ziformula=~SCM_Type+Period+(1|Site))
summary(glmtypevsaps22)
```

```
##  Family: truncated_nbinom2  ( log )
## Formula:          aps100 ~ SCM_Type + Period + (1 | Site)
## Zero inflation:          ~SCM_Type + Period + (1 | Site)
## Data: siteperiodaps22
## 
##      AIC      BIC   logLik deviance df.resid 
##   2077.8   2167.3  -1013.9   2027.8      241 
## 
## Random effects:
## 
## Conditional model:
##  Groups Name        Variance Std.Dev.
##  Site   (Intercept) 0.7736   0.8795  
## Number of obs: 266, groups:  Site, 30
## 
## Zero-inflation model:
##  Groups Name        Variance Std.Dev.
##  Site   (Intercept) 2.274    1.508   
## Number of obs: 266, groups:  Site, 30
## 
## Dispersion parameter for truncated_nbinom2 family (): 0.921 
## 
## Conditional model:
##             Estimate Std. Error z value Pr(>|z|)    
## (Intercept)   3.6677     0.5142   7.133 9.79e-13 ***
## SCM_TypeDP    1.0111     0.4870   2.076  0.03787 *  
## SCM_TypeRP    0.3804     0.4953   0.768  0.44239    
## Period2       1.6623     0.5188   3.205  0.00135 ** 
## Period3       0.5801     0.5060   1.146  0.25161    
## Period4       0.5528     0.5568   0.993  0.32074    
## Period5       1.4226     0.5197   2.738  0.00619 ** 
## Period6       1.1002     0.4984   2.207  0.02729 *  
## Period7       0.7805     0.5176   1.508  0.13158    
## Period8       1.1600     0.5221   2.222  0.02631 *  
## Period9       0.8270     0.5494   1.505  0.13223    
## ---
## Signif. codes:  0 '***' 0.001 '**' 0.01 '*' 0.05 '.' 0.1 ' ' 1
## 
## Zero-inflation model:
##             Estimate Std. Error z value Pr(>|z|)    
## (Intercept)   0.5366     0.7216   0.744 0.457100    
## SCM_TypeDP    1.4530     0.7945   1.829 0.067427 .  
## SCM_TypeRP    1.3796     0.8013   1.722 0.085105 .  
## Period2      -1.0814     0.6878  -1.572 0.115865    
## Period3      -1.6733     0.6769  -2.472 0.013433 *  
## Period4      -1.0807     0.6715  -1.609 0.107546    
## Period5      -2.7189     0.7182  -3.786 0.000153 ***
## Period6      -2.7189     0.7182  -3.786 0.000153 ***
## Period7      -2.0739     0.6877  -3.016 0.002565 ** 
## Period8      -1.6733     0.6769  -2.472 0.013433 *  
## Period9      -1.0192     0.6839  -1.490 0.136159    
## ---
## Signif. codes:  0 '***' 0.001 '**' 0.01 '*' 0.05 '.' 0.1 ' ' 1
```

```
Anova(glmtypevsaps22, component="cond") #only period is significant
```

```
## Analysis of Deviance Table (Type II Wald chisquare tests)
## 
## Response: aps100
##            Chisq Df Pr(>Chisq)  
## SCM_Type  4.3345  2    0.11449  
## Period   18.4852  8    0.01787 *
## ---
## Signif. codes:  0 '***' 0.001 '**' 0.01 '*' 0.05 '.' 0.1 ' ' 1
```

```
Anova(glmtypevsaps22, component="zi") #only period is significant
```

```
## Analysis of Deviance Table (Type II Wald chisquare tests)
## 
## Response: aps100
##            Chisq Df Pr(>Chisq)   
## SCM_Type  4.1595  2   0.124962   
## Period   23.1351  8   0.003195 **
## ---
## Signif. codes:  0 '***' 0.001 '**' 0.01 '*' 0.05 '.' 0.1 ' ' 1
```

```
emmeans(glmtypevsaps22, component="zi", pairwise~SCM_Type) #all are non-significant
```

```
## $emmeans
##  SCM_Type emmean    SE  df lower.CL upper.CL
##  CW       -1.023 0.567 241   -2.140   0.0929
##  DP        0.430 0.551 241   -0.656   1.5156
##  RP        0.356 0.558 241   -0.742   1.4547
## 
## Results are averaged over the levels of: Period 
## Results are given on the logit (not the response) scale. 
## Confidence level used: 0.95 
## 
## $contrasts
##  contrast estimate    SE  df t.ratio p.value
##  CW - DP   -1.4530 0.794 241  -1.829  0.1624
##  CW - RP   -1.3796 0.801 241  -1.722  0.1991
##  DP - RP    0.0734 0.782 241   0.094  0.9952
## 
## Results are averaged over the levels of: Period 
## Results are given on the log odds ratio (not the response) scale. 
## P value adjustment: tukey method for comparing a family of 3 estimates
```

```
emmeans(glmtypevsaps22, component="zi", pairwise~Period)
```

```
## $emmeans
##  Period emmean    SE  df lower.CL upper.CL
##  1       1.481 0.578 241    0.343    2.619
##  2       0.399 0.548 241   -0.680    1.479
##  3      -0.192 0.527 241   -1.230    0.845
##  4       0.400 0.529 241   -0.643    1.443
##  5      -1.238 0.562 241   -2.346   -0.131
##  6      -1.238 0.562 241   -2.346   -0.131
##  7      -0.593 0.534 241   -1.645    0.459
##  8      -0.192 0.527 241   -1.230    0.845
##  9       0.462 0.546 241   -0.614    1.537
## 
## Results are averaged over the levels of: SCM_Type 
## Results are given on the logit (not the response) scale. 
## Confidence level used: 0.95 
## 
## $contrasts
##  contrast           estimate    SE  df t.ratio p.value
##  Period1 - Period2  1.081444 0.688 241   1.572  0.8189
##  Period1 - Period3  1.673264 0.677 241   2.472  0.2510
##  Period1 - Period4  1.080673 0.672 241   1.609  0.7990
##  Period1 - Period5  2.718851 0.718 241   3.786  0.0059
##  Period1 - Period6  2.718851 0.718 241   3.786  0.0059
##  Period1 - Period7  2.073855 0.688 241   3.016  0.0690
##  Period1 - Period8  1.673264 0.677 241   2.472  0.2510
##  Period1 - Period9  1.019225 0.684 241   1.490  0.8592
##  Period2 - Period3  0.591821 0.648 241   0.913  0.9921
##  Period2 - Period4 -0.000771 0.649 241  -0.001  1.0000
##  Period2 - Period5  1.637407 0.682 241   2.402  0.2879
##  Period2 - Period6  1.637407 0.682 241   2.402  0.2879
##  Period2 - Period7  0.992411 0.656 241   1.514  0.8483
##  Period2 - Period8  0.591821 0.648 241   0.913  0.9921
##  Period2 - Period9 -0.062218 0.661 241  -0.094  1.0000
##  Period3 - Period4 -0.592592 0.632 241  -0.938  0.9906
##  Period3 - Period5  1.045587 0.659 241   1.587  0.8112
##  Period3 - Period6  1.045587 0.659 241   1.587  0.8112
##  Period3 - Period7  0.400590 0.635 241   0.631  0.9994
##  Period3 - Period8  0.000000 0.629 241   0.000  1.0000
##  Period3 - Period9 -0.654039 0.646 241  -1.012  0.9845
##  Period4 - Period5  1.638178 0.667 241   2.455  0.2596
##  Period4 - Period6  1.638178 0.667 241   2.455  0.2596
##  Period4 - Period7  0.993182 0.640 241   1.552  0.8294
##  Period4 - Period8  0.592592 0.632 241   0.938  0.9906
##  Period4 - Period9 -0.061447 0.646 241  -0.095  1.0000
##  Period5 - Period6  0.000000 0.678 241   0.000  1.0000
##  Period5 - Period7 -0.644996 0.661 241  -0.976  0.9877
##  Period5 - Period8 -1.045587 0.659 241  -1.587  0.8112
##  Period5 - Period9 -1.699626 0.682 241  -2.493  0.2406
##  Period6 - Period7 -0.644996 0.661 241  -0.976  0.9877
##  Period6 - Period8 -1.045587 0.659 241  -1.587  0.8112
##  Period6 - Period9 -1.699626 0.682 241  -2.493  0.2406
##  Period7 - Period8 -0.400590 0.635 241  -0.631  0.9994
##  Period7 - Period9 -1.054629 0.655 241  -1.611  0.7979
##  Period8 - Period9 -0.654039 0.646 241  -1.012  0.9845
## 
## Results are averaged over the levels of: SCM_Type 
## Results are given on the log odds ratio (not the response) scale. 
## P value adjustment: tukey method for comparing a family of 9 estimates
```

```
siteperiodaps22$pos<-ifelse(siteperiodaps22$aps100>0, siteperiodaps22$x, NA)
siteperiodaps22$logpos<-log10(siteperiodaps22$pos)
ggplot() +
  geom_boxplot(data=siteperiodaps22, aes(x=Period, y=logpos, color=SCM_Type)) +
  theme_classic() +
  scale_color_manual("SCM Type", values=type.colors, labels=c("Const. Wetland", "Detention Pond", "Retention Pond")) + 
  xlab("Period") + ylab(label=expression(Log["10"]*APS)) +ylim(-1,2)
```

```
## Warning: Removed 133 rows containing non-finite values (`stat_boxplot()`).
```

Compare mosquito species and total abundance with SCM type and environmental characteristics using Partial Least Squares Canonical Analyses

```
library(mixOmics)
```

```
## Warning: package 'mixOmics' was built under R version 4.1.2
```

```
## Loading required package: MASS
```

```
## Loading required package: lattice
```

```
## Warning: package 'lattice' was built under R version 4.1.2
```

```
## 
## Loaded mixOmics 6.18.1
## Thank you for using mixOmics!
## Tutorials: http://mixomics.org
## Bookdown vignette: https://mixomicsteam.github.io/Bookdown
## Questions, issues: Follow the prompts at http://mixomics.org/contact-us
## Cite us:  citation('mixOmics')
```

```
#2021 Data
scmdata21$RP <- ifelse(scmdata21$SCM_Type == 'RP', 1, 0) #create dummy variables for SCM types
scmdata21$CW <- ifelse(scmdata21$SCM_Type == 'CW', 1, 0)
scmdata21$DP <- ifelse(scmdata21$SCM_Type == 'DP', 1, 0)

pls21<-pls(scmdata21[c(7:13,26:28)], scmdata21[c(14,16:20,22,23,25)], mode = c("canonical"), ncomp = 2, scale = TRUE, max.iter = 100) #generate partial least squares canonical analysis
pls21
```

```
## 
## Call:
##  pls(X = scmdata21[c(7:13, 26:28)], Y = scmdata21[c(14, 16:20, 22, 23, 25)], ncomp = 2, scale = TRUE, mode = c("canonical"), max.iter = 100) 
## 
##  PLS with a 'canonical' mode with 2 PLS components. 
##  You entered data X of dimensions: 2096 10 
##  You entered data Y of dimensions: 2096 9 
## 
##  No variable selection. 
## 
##  Main numerical outputs: 
##  -------------------- 
##  loading vectors: see object$loadings 
##  variates: see object$variates 
##  variable names: see object$names 
## 
##  Functions to visualise samples: 
##  -------------------- 
##  plotIndiv, plotArrow 
## 
##  Functions to visualise variables: 
##  -------------------- 
##  plotVar, plotLoadings, network, cim
```

```
plotVar(pls21)
```

```
network(pls21, cutoff = 0.4, color.edge = color.spectral(2), lty.edge = c("solid", "dashed"), lwd.edge = 2) #Analysis shows correlations greater than |0.40|
```

```
hm.mat.pls21<-network(pls21)$M #Extract the similarity matrix from the PLSCA
```

```
heatmap(hm.mat.pls21, scale="none", col=c(hm.colors))
```

```
#2022 Data
scmdata22$RP <- ifelse(scmdata22$SCM_Type == 'RP', 1, 0) #create dummy variables for SCM types
scmdata22$CW <- ifelse(scmdata22$SCM_Type == 'CW', 1, 0)
scmdata22$DP <- ifelse(scmdata22$SCM_Type == 'DP', 1, 0)

pls22<-pls(scmdata22[c(7:13,26:28)], scmdata22[c(14:23,25)], mode = c("canonical"), ncomp = 2, scale = TRUE, max.iter = 100)
pls22
```

```
## 
## Call:
##  pls(X = scmdata22[c(7:13, 26:28)], Y = scmdata22[c(14:23, 25)], ncomp = 2, scale = TRUE, mode = c("canonical"), max.iter = 100) 
## 
##  PLS with a 'canonical' mode with 2 PLS components. 
##  You entered data X of dimensions: 2160 10 
##  You entered data Y of dimensions: 2160 11 
## 
##  No variable selection. 
## 
##  Main numerical outputs: 
##  -------------------- 
##  loading vectors: see object$loadings 
##  variates: see object$variates 
##  variable names: see object$names 
## 
##  Functions to visualise samples: 
##  -------------------- 
##  plotIndiv, plotArrow 
## 
##  Functions to visualise variables: 
##  -------------------- 
##  plotVar, plotLoadings, network, cim
```

```
plotVar(pls22)
```

```
network(pls22, cutoff = 0.4, color.edge = color.spectral(2), lty.edge = c("solid", "dashed"), lwd.edge = 2) #Analysis shows correlations greater than |0.40|
```

```
hm.mat.pls22<-network(pls22)$M
```

```
heatmap(hm.mat.pls22, scale="none", col=hm.colors)
```

Indicator species analysis of SCM types

```
library(indicspecies)
```

```
## Warning: package 'indicspecies' was built under R version 4.1.3
```

```
## Loading required package: permute
```

```
summary(multipatt(x=sitetotals21[,2:11], cluster=sitetotals21$SCM_Type)) #2021 indicator species analysis
```

```
## 
##  Multilevel pattern analysis
##  ---------------------------
## 
##  Association function: IndVal.g
##  Significance level (alpha): 0.05
## 
##  Total number of species: 10
##  Selected number of species: 3 
##  Number of species associated to 1 group: 1 
##  Number of species associated to 2 groups: 2 
## 
##  List of species associated to each combination: 
## 
##  Group CW  #sps.  1 
##        stat p.value  
## Ur.sa 0.678   0.015 *
## 
##  Group CW+RP  #sps.  2 
##        stat p.value  
## An.   0.880   0.025 *
## Cx.er 0.857   0.015 *
## ---
## Signif. codes:  0 '***' 0.001 '**' 0.01 '*' 0.05 '.' 0.1 ' ' 1
```

```
summary(multipatt(x=sitetotals22[,2:11], cluster=sitetotals22$SCM_Type)) #2022 indicator species analysis
```

```
## 
##  Multilevel pattern analysis
##  ---------------------------
## 
##  Association function: IndVal.g
##  Significance level (alpha): 0.05
## 
##  Total number of species: 10
##  Selected number of species: 2 
##  Number of species associated to 1 group: 1 
##  Number of species associated to 2 groups: 1 
## 
##  List of species associated to each combination: 
## 
##  Group CW  #sps.  1 
##        stat p.value  
## Cx.sa 0.698   0.025 *
## 
##  Group CW+RP  #sps.  1 
##        stat p.value  
## Cx.er 0.831   0.015 *
## ---
## Signif. codes:  0 '***' 0.001 '**' 0.01 '*' 0.05 '.' 0.1 ' ' 1
```

Alpha diversity of mosquitoes among SCM types

```
library(vegan)
```

```
## This is vegan 2.5-7
```

```
library(ggpubr)

#2021 Data
sitetotals21.nozero<-sitetotals21[sitetotals21$Mosq!=0,] #Need to remove sites with no mosquitoes

#Generate alpha diversity values for each site
sitetotals21.nozero$shannon<-diversity(sitetotals21.nozero[,2:11], index="shannon") #Shannon index
sitetotals21.nozero$richness<-apply(sitetotals21.nozero[,2:11]>0, 1, sum) #Species richness
sitetotals21.nozero$evenness<-sitetotals21.nozero$shannon/log(sitetotals21.nozero$richness) #Shannon evenness
print(sitetotals21.nozero)
```

```
##    Site Ae.ve Ps.fe Cx.te Cx.er Cx.pi Cx.re Cx.sa Cs.in An. Ur.sa Unidentified
## 1  DP11     6     0    27     0   143     1     0     0   7     0            9
## 2  DP12     0     0     0     0     0     0     0     0   9     0            0
## 3  DP13     1     0     0     0     0     0     0     0   1     0            0
## 5  DP15     0     0     0     0     0     0     0     0   3     0            0
## 7  DP18     0     0     4    19     0     0     0     0  13     0            0
## 8  DP20   183     0     0     0    17     0     0     0   0     0            0
## 10  DP9     0     0     1     0     0     0     0     0   3     0            0
## 11  P12     0     0   349    10     0     0     0     0  23     4            0
## 12  P14     0     0     5    34     0     0     0     0  27     0            1
## 13  P15     0     0     0     5     0     0     0     0   0     0            0
## 15   P2     0     0     0     0     0     0     0     0   2     0            1
## 16  P21     4     0     2     9     0     0     0     0   8     0            0
## 17  P27     0     0     1    27     0     0     0     0  46     0            0
## 18  P28     0     0     8    18     0     0     0     0  17     0            0
## 20   P7     0     0     0    61     0     0     0     0  51     0            0
## 21   W1    79     0    52     3     0     0     0     0  10     2            4
## 22  W10     0     0    10     4     0     0     0     0  57     0            1
## 23  W11     4     0    16     0     0     0     0     0   5     6            0
## 24  W12   143     0    65    29     1     0     0     0  39    24            0
## 25  W13     5     0     7     3     0     0     0     0  18     0            0
## 26  W20     0     0   511     1   211    10    24     0  19    11            5
## 27   W4     0     0    15     6     1     0     0     0  15     0            0
## 28   W5     0     0     0     1     1     0     0     0  12     0            1
## 29   W6     0     0   181     1     5     0     0     0   7     3            7
## 30   W9     0     0    22     3     0     0     0     0  13     0            0
##    Mosq SCM_Type   shannon richness  evenness
## 1   193       DP 0.7418580        5 0.4609423
## 2     9       DP 0.0000000        1       NaN
## 3     2       DP 0.6931472        2 1.0000000
## 5     3       DP 0.0000000        1       NaN
## 7    36       DP 0.9492451        3 0.8640401
## 8   200       DP 0.2908144        2 0.4195565
## 10    4       DP 0.5623351        2 0.8112781
## 11  388       RP 0.4011547        4 0.2893720
## 12   67       RP 0.9028208        3 0.8217829
## 13    5       RP 0.0000000        1       NaN
## 15    3       RP 0.0000000        1       NaN
## 16   23       RP 1.2510583        4 0.9024478
## 17   74       RP 0.7215637        3 0.6567956
## 18   43       RP 1.0442966        3 0.9505597
## 20  112       RP 0.6891559        2 0.9942418
## 21  150       CW 1.0222434        5 0.6351555
## 22   72       CW 0.6144411        3 0.5592884
## 23   31       CW 1.2177181        4 0.8783979
## 24  301       CW 1.3953969        6 0.7787858
## 25   33       CW 1.1634443        4 0.8392476
## 26  792       CW 0.9533016        7 0.4899001
## 27   37       CW 1.1246459        4 0.8112606
## 28   15       CW 0.5091373        3 0.4634368
## 29  204       CW 0.3801968        5 0.2362296
## 30   38       CW 0.8838200        3 0.8044877
```

```
#Test Shannon diversity
aggregate(sitetotals21.nozero[,15], list(sitetotals21.nozero$SCM_Type), FUN=mean); aggregate(sitetotals21.nozero[,15], list(sitetotals21.nozero$SCM_Type), FUN=sd) #View mean and standard deviation for each SCM Type
```

```
##   Group.1         x
## 1      CW 0.9264345
## 2      DP 0.4624857
## 3      RP 0.6262563
```

```
##   Group.1         x
## 1      CW 0.3302865
## 2      DP 0.3731432
## 3      RP 0.4613223
```

```
ggboxplot(sitetotals21.nozero, x="SCM_Type", y="shannon") #View box plot
```

```
kruskal.test(sitetotals21.nozero$shannon~sitetotals21.nozero$SCM_Type) #Means not significant
```

```
## 
##  Kruskal-Wallis rank sum test
## 
## data:  sitetotals21.nozero$shannon by sitetotals21.nozero$SCM_Type
## Kruskal-Wallis chi-squared = 4.912, df = 2, p-value = 0.08578
```

```
#Test species richness
aggregate(sitetotals21.nozero[,16], list(sitetotals21.nozero$SCM_Type), FUN=mean); aggregate(sitetotals21.nozero[,16], list(sitetotals21.nozero$SCM_Type), FUN=sd)
```

```
##   Group.1        x
## 1      CW 4.400000
## 2      DP 2.285714
## 3      RP 2.625000
```

```
##   Group.1        x
## 1      CW 1.349897
## 2      DP 1.380131
## 3      RP 1.187735
```

```
ggboxplot(sitetotals21.nozero, x="SCM_Type", y="richness")
```

```
kruskal.test(sitetotals21.nozero$richness~sitetotals21.nozero$SCM_Type) #Means are significantly different
```

```
## 
##  Kruskal-Wallis rank sum test
## 
## data:  sitetotals21.nozero$richness by sitetotals21.nozero$SCM_Type
## Kruskal-Wallis chi-squared = 9.299, df = 2, p-value = 0.009566
```

```
pairwise.wilcox.test(sitetotals21.nozero$richness, sitetotals21.nozero$SCM_Type, p.adjust.method="bonferroni") #Check pairwise comparison
```

```
## Warning in wilcox.test.default(xi, xj, paired = paired, ...): cannot compute
## exact p-value with ties

## Warning in wilcox.test.default(xi, xj, paired = paired, ...): cannot compute
## exact p-value with ties

## Warning in wilcox.test.default(xi, xj, paired = paired, ...): cannot compute
## exact p-value with ties
```

```
## 
##  Pairwise comparisons using Wilcoxon rank sum test with continuity correction 
## 
## data:  sitetotals21.nozero$richness and sitetotals21.nozero$SCM_Type 
## 
##    CW    DP   
## DP 0.030 -    
## RP 0.052 1.000
## 
## P value adjustment method: bonferroni
```

```
#Test Shannon evenness
aggregate(sitetotals21.nozero[,17], list(sitetotals21.nozero$SCM_Type), FUN=mean, na.rm=T); aggregate(sitetotals21.nozero[,17], list(sitetotals21.nozero$SCM_Type), FUN=sd, na.rm=T)
```

```
##   Group.1         x
## 1      CW 0.6496190
## 2      DP 0.7111634
## 3      RP 0.7692000
```

```
##   Group.1         x
## 1      CW 0.2093717
## 2      DP 0.2571313
## 3      RP 0.2634102
```

```
ggboxplot(sitetotals21.nozero, x="SCM_Type", y="evenness")
```

```
## Warning: Removed 4 rows containing non-finite values (`stat_boxplot()`).
```

```
kruskal.test(sitetotals21.nozero$evenness~sitetotals21.nozero$SCM_Type) #Means not significant
```

```
## 
##  Kruskal-Wallis rank sum test
## 
## data:  sitetotals21.nozero$evenness by sitetotals21.nozero$SCM_Type
## Kruskal-Wallis chi-squared = 1.9706, df = 2, p-value = 0.3733
```

```
#2022 Data
sitetotals22.nozero<-sitetotals22[sitetotals22$Mosq!=0,] #Need to remove sites with no mosquitoes

#Generate alpha diversity values for each site
sitetotals22.nozero$shannon<-diversity(sitetotals22.nozero[,2:11], index="shannon") #Shannon index
sitetotals22.nozero$richness<-apply(sitetotals22.nozero[,2:11]>0, 1, sum) #Species richness
sitetotals22.nozero$evenness<-sitetotals22.nozero$shannon/log(sitetotals22.nozero$richness) #Shannon evenness
print(sitetotals22.nozero)
```

```
##    Site Ae.ve Ps.fe Cx.te Cx.er Cx.pi Cx.re Cx.sa Cs.in An. Ur.sa Unidentified
## 1  DP11    25     0     9     0   383     8     0     0   7     0            0
## 2  DP12     0     0     0     0     0     0     0     0   7     0            0
## 3  DP13     0     0     2     0     0     0     0     0  13     0            0
## 4  DP14     0     0     0     5     0     0     0     0  15     0            0
## 6  DP17     9     0     0     0    28    14     2     0   2     0            7
## 7  DP18     0     0     0     3     0     0     0     0   9     0            0
## 8  DP20    29     0     0     0   164     1     2     0   5     0            0
## 9   DP4     0     0     0     0    36     0     0     0   1     0            0
## 10  DP9     0     0     8     0     0     0     0     0   0     0            0
## 11  P12     0     0    20     8     2    95     0     0  12     0            0
## 12  P14     0     0     1   142     0     0     0     0  60    10            0
## 14  P19     0     0     0     0     2     0     0     0   0     0            0
## 15   P2     0     0     6     0     0     0     0     0   3     1            0
## 16  P21     0     0     2    16     7     0     0     0  18     1            0
## 17  P27     0     0     1   169     0     0     0     0  33     0            3
## 18  P28     0     0     1   104     0     0     0     0  65     0            1
## 20   P7     0     0    12    60     0     0     0     0  47     0            0
## 21   W1     0     0     2     4    16     0     8     0  26     1            0
## 22  W10     0     0     0    16     0     0     0     0   1     0            0
## 23  W11     8     0     0     0     1     0     0     0   2     0            1
## 24  W12     4     0     0     1     0     0     1     0  10     1            0
## 25  W13     0     0     1     6     0     0     0     0  16     0            1
## 26  W20    59     2   408     0    77   102   137    14  25     1            1
## 27   W4     0     0    19     6     1     0     0     0  19     0            0
## 28   W5     0     0     6    15     0     0     3     0  65     0            0
## 29   W6    23     0    51     7    32     0     3     0   5     1            2
## 30   W9     0     0    12     1     0     0     0     0   6     1            1
##    Mosq SCM_Type   shannon richness  evenness
## 1   432       DP 0.4929598        5 0.3062931
## 2     7       DP 0.0000000        1       NaN
## 3    15       DP 0.3926745        2 0.5665095
## 4    20       DP 0.5623351        2 0.8112781
## 6    62       DP 1.2292216        5 0.7637583
## 7    12       DP 0.5623351        2 0.8112781
## 8   201       DP 0.6094584        5 0.3786778
## 9    37       DP 0.1242508        2 0.1792561
## 10    8       DP 0.0000000        1       NaN
## 11  137       RP 0.9756477        5 0.6062040
## 12  213       RP 0.7959684        4 0.5741698
## 14    2       RP 0.0000000        1       NaN
## 15   10       RP 0.8979457        3 0.8173454
## 16   44       RP 1.2524674        5 0.7782018
## 17  206       RP 0.4741043        3 0.4315484
## 18  171       RP 0.6984348        3 0.6357427
## 20  119       RP 0.9435227        3 0.8588314
## 21   57       CW 1.3651752        6 0.7619188
## 22   17       CW 0.2237181        2 0.3227570
## 23   12       CW 0.7595474        3 0.6913698
## 24   17       CW 1.1525646        5 0.7161287
## 25   24       CW 0.7393213        3 0.6729593
## 26  826       CW 1.5126751        9 0.6884481
## 27   45       CW 1.0813461        4 0.7800263
## 28   89       CW 0.8256890        4 0.5956087
## 29  124       CW 1.4555996        7 0.7480302
## 30   21       CW 0.9672604        4 0.6977309
```

```
#Test Shannon diversity
aggregate(sitetotals22.nozero[,15], list(sitetotals22.nozero$SCM_Type), FUN=mean); aggregate(sitetotals22.nozero[,15], list(sitetotals22.nozero$SCM_Type), FUN=sd) #View mean and standard deviation for each SCM Type
```

```
##   Group.1         x
## 1      CW 1.0082897
## 2      DP 0.4414706
## 3      RP 0.7547614
```

```
##   Group.1         x
## 1      CW 0.3933907
## 2      DP 0.3829884
## 3      RP 0.3790851
```

```
ggboxplot(sitetotals22.nozero, x="SCM_Type", y="shannon") #View box plot
```

```
kruskal.test(sitetotals22.nozero$shannon~sitetotals22.nozero$SCM_Type) #Means are significantly different
```

```
## 
##  Kruskal-Wallis rank sum test
## 
## data:  sitetotals22.nozero$shannon by sitetotals22.nozero$SCM_Type
## Kruskal-Wallis chi-squared = 8.2749, df = 2, p-value = 0.01596
```

```
pairwise.wilcox.test(sitetotals22.nozero$shannon, sitetotals22.nozero$SCM_Type, p.adjust.method="bonferroni") #Check pairwise comparison
```

```
## Warning in wilcox.test.default(xi, xj, paired = paired, ...): cannot compute
## exact p-value with ties

## Warning in wilcox.test.default(xi, xj, paired = paired, ...): cannot compute
## exact p-value with ties
```

```
## 
##  Pairwise comparisons using Wilcoxon rank sum test with continuity correction 
## 
## data:  sitetotals22.nozero$shannon and sitetotals22.nozero$SCM_Type 
## 
##    CW    DP   
## DP 0.024 -    
## RP 0.609 0.274
## 
## P value adjustment method: bonferroni
```

```
#Test species richness
aggregate(sitetotals22.nozero[,16], list(sitetotals22.nozero$SCM_Type), FUN=mean); aggregate(sitetotals22.nozero[,16], list(sitetotals22.nozero$SCM_Type), FUN=sd)
```

```
##   Group.1        x
## 1      CW 4.700000
## 2      DP 2.777778
## 3      RP 3.375000
```

```
##   Group.1        x
## 1      CW 2.110819
## 2      DP 1.715938
## 3      RP 1.302470
```

```
ggboxplot(sitetotals22.nozero, x="SCM_Type", y="richness")
```

```
kruskal.test(sitetotals22.nozero$richness~sitetotals22.nozero$SCM_Type) #Means not significant
```

```
## 
##  Kruskal-Wallis rank sum test
## 
## data:  sitetotals22.nozero$richness by sitetotals22.nozero$SCM_Type
## Kruskal-Wallis chi-squared = 4.4529, df = 2, p-value = 0.1079
```

```
#Test Shannon evenness
aggregate(sitetotals22.nozero[,17], list(sitetotals22.nozero$SCM_Type), FUN=mean, na.rm=T); aggregate(sitetotals22.nozero[,17], list(sitetotals22.nozero$SCM_Type), FUN=sd, na.rm=T)
```

```
##   Group.1         x
## 1      CW 0.6674978
## 2      DP 0.5452930
## 3      RP 0.6717205
```

```
##   Group.1         x
## 1      CW 0.1318228
## 2      DP 0.2609400
## 3      RP 0.1529384
```

```
ggboxplot(sitetotals22.nozero, x="SCM_Type", y="evenness")
```

```
## Warning: Removed 3 rows containing non-finite values (`stat_boxplot()`).
```

```
kruskal.test(sitetotals22.nozero$evenness~sitetotals22.nozero$SCM_Type) #Means not significant
```

```
## 
##  Kruskal-Wallis rank sum test
## 
## data:  sitetotals22.nozero$evenness by sitetotals22.nozero$SCM_Type
## Kruskal-Wallis chi-squared = 0.81092, df = 2, p-value = 0.6667
```

Beta diversity of mosquitoes among SCM sites

```
library(pairwiseAdonis)
```

```
## Loading required package: cluster
```

```
#2021 Data
sitetotals21.nozero.matrix<-as.matrix(sitetotals21.nozero[,2:11]) #need matrix format for NMDS; matrix only including community data
nmds21<-metaMDS(sitetotals21.nozero.matrix, distance="bray") #Generate NMDS
```

```
## Square root transformation
## Wisconsin double standardization
## Run 0 stress 0.09968259 
## Run 1 stress 0.09968259 
## ... New best solution
## ... Procrustes: rmse 1.125899e-05  max resid 4.003061e-05 
## ... Similar to previous best
## Run 2 stress 0.09968255 
## ... New best solution
## ... Procrustes: rmse 0.0003546585  max resid 0.00131705 
## ... Similar to previous best
## Run 3 stress 0.09968249 
## ... New best solution
## ... Procrustes: rmse 0.0002638756  max resid 0.0009748672 
## ... Similar to previous best
## Run 4 stress 0.1344696 
## Run 5 stress 0.2227763 
## Run 6 stress 0.09968253 
## ... Procrustes: rmse 4.496043e-05  max resid 0.0001695881 
## ... Similar to previous best
## Run 7 stress 0.1993041 
## Run 8 stress 0.09968251 
## ... Procrustes: rmse 2.633336e-05  max resid 9.724468e-05 
## ... Similar to previous best
## Run 9 stress 0.1344696 
## Run 10 stress 0.09968258 
## ... Procrustes: rmse 8.430799e-05  max resid 0.0003165103 
## ... Similar to previous best
## Run 11 stress 0.09968277 
## ... Procrustes: rmse 0.000193891  max resid 0.0007226838 
## ... Similar to previous best
## Run 12 stress 0.09968271 
## ... Procrustes: rmse 0.0001645472  max resid 0.0006142802 
## ... Similar to previous best
## Run 13 stress 0.1344694 
## Run 14 stress 0.1652107 
## Run 15 stress 0.09968281 
## ... Procrustes: rmse 0.0002146393  max resid 0.0007982052 
## ... Similar to previous best
## Run 16 stress 0.09968251 
## ... Procrustes: rmse 0.0002146656  max resid 0.0007866555 
## ... Similar to previous best
## Run 17 stress 0.09968244 
## ... New best solution
## ... Procrustes: rmse 0.0001044317  max resid 0.0003820622 
## ... Similar to previous best
## Run 18 stress 0.0996825 
## ... Procrustes: rmse 0.0001161572  max resid 0.0004326984 
## ... Similar to previous best
## Run 19 stress 0.1344696 
## Run 20 stress 0.09968244 
## ... New best solution
## ... Procrustes: rmse 2.749438e-06  max resid 7.084735e-06 
## ... Similar to previous best
## *** Solution reached
```

```
#Visualize NMDS in ggplot
nmds21.ds<-as.data.frame(scores(nmds21)) #Extract coordinates of Sites
nmds21.ds$site<-sitetotals21.nozero$Site; nmds21.ds$type<-sitetotals21.nozero$SCM_Type
ggplot(nmds21.ds, aes(NMDS1, NMDS2)) +
  theme_classic() +
  geom_point(size=4, aes(color=type)) + stat_ellipse(aes(color=type)) + scale_color_manual("SCM Type", values=type.colors, labels=c("Const. Wetland","Detention Pond", "Retention Pond"))
```

```
#Test if SCM Types harbor different beta diversity
adonis(sitetotals21.nozero.matrix~sitetotals21.nozero$SCM_Type, perm=999, method="bray") #Centroids are significantly different
```

```
## 
## Call:
## adonis(formula = sitetotals21.nozero.matrix ~ sitetotals21.nozero$SCM_Type,      permutations = 999, method = "bray") 
## 
## Permutation: free
## Number of permutations: 999
## 
## Terms added sequentially (first to last)
## 
##                              Df SumsOfSqs MeanSqs F.Model      R2 Pr(>F)  
## sitetotals21.nozero$SCM_Type  2    1.2472 0.62360  2.1174 0.16142  0.013 *
## Residuals                    22    6.4794 0.29452         0.83858         
## Total                        24    7.7266                 1.00000         
## ---
## Signif. codes:  0 '***' 0.001 '**' 0.01 '*' 0.05 '.' 0.1 ' ' 1
```

```
pairwise.adonis(sitetotals21.nozero.matrix, sitetotals21.nozero$SCM_Type) #No pairs are significantly different
```

```
##      pairs Df SumsOfSqs  F.Model        R2 p.value p.adjusted sig
## 1 DP vs RP  1 0.6366622 1.980368 0.1321976   0.067      0.201    
## 2 DP vs CW  1 0.7026259 2.381076 0.1369924   0.017      0.051    
## 3 RP vs CW  1 0.5380725 1.977716 0.1100093   0.071      0.213
```

```
#2022 Data
sitetotals22.nozero.matrix<-as.matrix(sitetotals22.nozero[,2:11]) #need matrix format for NMDS; matrix only including community data
nmds22<-metaMDS(sitetotals22.nozero.matrix, distance="bray") #Generate NMDS
```

```
## Square root transformation
## Wisconsin double standardization
## Run 0 stress 0.1179208 
## Run 1 stress 0.1259966 
## Run 2 stress 0.1442765 
## Run 3 stress 0.1384494 
## Run 4 stress 0.1384493 
## Run 5 stress 0.1179208 
## ... New best solution
## ... Procrustes: rmse 9.799615e-06  max resid 3.349594e-05 
## ... Similar to previous best
## Run 6 stress 0.1183811 
## ... Procrustes: rmse 0.07526973  max resid 0.1922142 
## Run 7 stress 0.1645974 
## Run 8 stress 0.1578616 
## Run 9 stress 0.1353357 
## Run 10 stress 0.163584 
## Run 11 stress 0.1384493 
## Run 12 stress 0.1267026 
## Run 13 stress 0.1353358 
## Run 14 stress 0.1264168 
## Run 15 stress 0.1260928 
## Run 16 stress 0.1179209 
## ... Procrustes: rmse 0.0001298818  max resid 0.0005001639 
## ... Similar to previous best
## Run 17 stress 0.1179208 
## ... New best solution
## ... Procrustes: rmse 1.51326e-05  max resid 6.456378e-05 
## ... Similar to previous best
## Run 18 stress 0.139423 
## Run 19 stress 0.1259967 
## Run 20 stress 0.1398359 
## *** Solution reached
```

```
#Visualize NMDS in ggplot
nmds22.ds<-as.data.frame(scores(nmds22)) #Extract coordinates of Sites
nmds22.ds$site<-sitetotals22.nozero$Site; nmds22.ds$type<-sitetotals22.nozero$SCM_Type
ggplot(nmds22.ds, aes(NMDS1, NMDS2)) +
  theme_classic() +
  geom_point(size=4, aes(color=type)) + stat_ellipse(aes(color=type)) + scale_color_manual("SCM Type", values=type.colors, labels=c("Const. Wetland","Detention Pond", "Retention Pond"))
```

```
## Warning in MASS::cov.trob(data[, vars]): Probable convergence failure
```

```
#Test if SCM Types harbor different beta diversity
adonis(sitetotals22.nozero.matrix~sitetotals22.nozero$SCM_Type, perm=999, method="bray") #Centroids are significantly different
```

```
## 
## Call:
## adonis(formula = sitetotals22.nozero.matrix ~ sitetotals22.nozero$SCM_Type,      permutations = 999, method = "bray") 
## 
## Permutation: free
## Number of permutations: 999
## 
## Terms added sequentially (first to last)
## 
##                              Df SumsOfSqs MeanSqs F.Model      R2 Pr(>F)  
## sitetotals22.nozero$SCM_Type  2    1.1562 0.57810  1.8077 0.13092  0.026 *
## Residuals                    24    7.6750 0.31979         0.86908         
## Total                        26    8.8312                 1.00000         
## ---
## Signif. codes:  0 '***' 0.001 '**' 0.01 '*' 0.05 '.' 0.1 ' ' 1
```

```
pairwise.adonis(sitetotals22.nozero.matrix, sitetotals22.nozero$SCM_Type) #No pairs are significantly different
```

```
##      pairs Df SumsOfSqs  F.Model         R2 p.value p.adjusted sig
## 1 DP vs RP  1 0.8816758 2.649783 0.15013122   0.014      0.042   .
## 2 DP vs CW  1 0.3563069 1.098819 0.06071219   0.322      0.966    
## 3 RP vs CW  1 0.5254909 1.734858 0.09782192   0.076      0.228
```
