# Supplementary material for: Mosquito abundance and diversity in central Ohio, USA vary among stormwater wetlands, retention ponds, and detention ponds and their associated environmental parameters
Source: PLoS One. 2024 Jun 25;19(6):e0305399. doi: 10.1371/journal.pone.0305399 (PMC11198753; doi:10.1371/journal.pone.0305399)
Supplement: Attachment — Submitted filename: Response to Reviewers.docx [file pone.0305399.s005.docx]

We thank the authors for their thoughtful reviews and helpful comments, the manuscript is improved after making their suggested changes. We have respond to each reviewer comment individually below. Our responses are written in red – note that all line number references refer to the Revised Manuscript with Track Changes

Reviewer #1:

Barcoding of the mt COI was done. Please upload your sequences to GenBank and provide IDs.

Sequences have been uploaded to GenBank. GenBank identifiers and accession numbers are provided in the revised S3 File.

Please include USA in the title.

Text edited as recommended

Change Cx. pipiens to Cx. pipiens s.l. or Cx. pipiens complex

While we recognize the difficulty in identification of the species in the *Cx. pipiens* complex using the methods we have chosen (morphological identity of larvae and COI barcoding), the only member of the *Cx. pipiens* complex known from the study region is *Cx. pipiens* (Harrison et al. 2016, Darsie and Ward 2005, Gorris et al. 2021). The “molestus” form/ecotype is known from this region, however, it is not currently recognized as a separate species (Harbach 2024)*.* Therefore, all specimens found in this study are expected to be true *Culex pipiens* specimens, and we feel that broadening this taxon identification is inappropriate and may lead to unnecessary confusion regarding the identity of our specimens (e.g., if readers assume our specimens include *Culex quinquefasciatus* specimens). We therefore respectfully request to maintain our nomenclature as currently written.

Harbach R. Mosquito taxonomic inventory. Mosquito Taxonomic Inventory; 2024. Available: <https://mosquito-taxonomic-inventory.myspecies.info/valid-species-list>

Gorris ME, Bartlow AW, Temple SD, Romero-Alvarez D, Shutt DP, Fair JM, et al. Updated distribution maps of predominant *Culex* mosquitoes across the Americas. Parasites & Vectors. 2021;14: 547. doi:[10.1186/s13071-021-05051-3](https://doi.org/10.1186/s13071-021-05051-3)

Darsie RF, Ward RA. Identification and geographical distribution of the mosquitoes of North America, north of Mexico. Gainesville, FL, USA: University Press of Florida; 2005.

Harrison BA, Byrd BD, Sither CB, Whitt PB. The mosquitoes of the Mid-Atlantic region: an identification guide. Cullowhee, N.C.: Western Carolina University; 2016.

L53: only pathogens can be spread - not diseases

Replaced “diseases” with “pathogens”

L 62: Point afer (6)

Text edited as recommended

L 147 and entire manuscript: Space after SI Unit (e.g. 50 cm)

Text edited as recommended

Line 176, 411, 412: Diseases cannot be transmitted

Changed to “pathogens”

L 326: There are differences at same sampling locations in two years - different species - please discuss

Text added 451-460 to discuss some of the different patterns we see among species, years, and SCM types.

Line 427: Malaria is the disease caused by Plasmodium spp.

Changed wording to directly reference transmission of *Plasmodium*

Line 499: Point after spp.

Text edited as recommended

Approximately 25 years ago, engineered structures designed to manage stormwater flows, recharge groundwater, capture trash, sediment, or other target pollutants began to be referred to collectively as “Best Management Practices”. Unfortunately, other actions related to stormwater and stormwater runoff also fell under the “BMP” umbrella, from policies to street sweeping to vegetation management. This was unnecessarily confusing, and fortunately the stormwater community has moved away from using this generic term when referring to engineered stormwater structures and replaced it with more specific names that define the category such as “trash capture devices”, “ground water recharge basins”, “constructed stormwater treatment wetlands”, etc.

With that said, I feel that your decision to use “Stormwater Control Measures – SCM” is similarly vague, like BMP, since “measures” here again could be any number of things including policy, actions, or structures. I strongly recommend you consider using a name that better indicates that these are engineered structures, e.g., Stormwater Management Structures; Stormwater Control Structures, etc.

We appreciate this thoughtful comment and the reviewer’s perspective. We agree that there is confusion over the usage of the name, and we also debated the correct terminology for some time. However, after consultation with our co-authors who work in stormwater engineering (Drs. Winston and Tyrpak), we feel Stormwater Control Measures is still the best term for our manuscript. The terminology surrounding these structures is reviewed extensively in Fletcher et al. 2013. Of note, they mention that “the US National Research Council of the National Academies of Engineering and Science commissioned a comprehensive review of stormwater management practice (National Research Council, 2008). One of the key findings was a universal agreement to abandon the term BMP in favour of stormwater control measure (SCM), to refer to both structural (e.g. bioretention systems) and non-structural (e.g. residential downspout disconnection programmes) control measures.” To the best of our knowledge, using SCM to refer to retention ponds, detention ponds, and constructed wetlands is standard in civil engineering literature, at least in the US, and we do not feel that our manuscript is an appropriate platform to challenge this paradigm. However, we recognize and agree with the issues raised by the reviewer, therefore, to help alleviate confusion, we added text to our definition of SCM (lines 68-69) to clarify that we are referring to the structural basins that store water specifically.

Fletcher TD, Shuster W, Hunt WF, Ashley R, Butler D, Arthur S, et al. SUDS, LID, BMPs, WSUD and more – The evolution and application of terminology surrounding urban drainage. Urban Water J. 2015;12: 525–542. doi:10.1080/1573062X.2014.916314.

The design and proper operation of any stormwater management device / structure falls apart without adequate maintenance. However, maintenance schedules and physical maintenance requirements are not universal and usually have to be considered at a minimum based on the category of a structure (e.g., detention basins), but more often on a site per site basis depending on the size of the structure, the amount of natural and urban generated (non-stormwater) runoff, trash and sediment loads entering, etc.

You tease us with the importance of maintenance in 3 areas of the manuscript: the introduction (lines 123-25), in the discussion opening paragraph (lines 393-394), and in the concluding paragraph of the paper (line 521). I would like to see a minimum of some thoughts on how the results of this study might inform future maintenance schedules, activities, and objectives (at least for the local study area). We know from experience that sediment loading can have severe impacts on operation of the structure and mosquito production. We know that steep sides are often not utilized by engineers due to public safety concerns. We know that design vegetation almost never holds and is eventually replaced by monocultures of Typha. There are so many issues related to design and maintenance. Please explore some ideas on how mosquito production could be better controlled / minimized for both nuisance biting and disease management. Why is maintenance important? How do requirements differ among the 3 categories of structures in your study?
Thank you for your input on this matter. We have expanded our discussion to include some of these points in the paragraph at lines 473-495 and lines 534-539.

A few editing and suggested odds and ends:

o I think you should consider revising the title of the paper. In particular, the “as well as associated environmental parameters” feels like an afterthought.

Changed to “as well as” to “and their” to avoid devaluing the importance of the environmental parameters

o Line 41. Replace determine with elucidate
Text edited as recommended

o Line 57. YF and malaria are no longer endemic in the USA. Revise to “Although diseases such as YF and malaria….”
Text edited as recommended

o Line 60. Revise sentence "Mosquitoes in the US can transmit lethal….”
Text edited as recommended

o Line 77. Consider including “available land / real estate” to this list
Text edited as recommended

o Lines 121-123. This sentence belongs in the Discussion

Moved line to discussion at line 409

o Line 130. “categories” is probably a better choice over “types”
While we are essentially categorizing the sites in this study, each category is a defined SCM “type” with each type sharing standard characteristics as defined in the introduction. We feel the term “type” more appropriately fits with the other contexts that this term is used in the introduction and discussion. The term “category” could apply to this sentence specifically, however, since this term is repeated throughout the study (including as a specific model factor name in the analysis), we feel that keeping the same terminology throughout minimizes confusion.

o Line 176. Remember that mosquitoes do not transmit “diseases”, they transmit pathogens.
Replaced “diseases” with “pathogens”

o Line 395. I think “harmful” should not be used when referring to certain species of mosquitoes, but rather “mosquitoes of public health significance or concern”
Text edited as recommended

o Line 511. As I mentioned above, we would love to see all basins built with steep sides to limit vegetation growth / shallow areas, and therefore reduce available larval habitat, but civil engineers often have to consider public safety (i.e. drownings) when designing and building stormwater control structures, which is why we don’t often see the steep sides that we wish for.

Added text to line 534-539 to address this.

o Line 516. I suggest you look at Bill Walton’s paper (UCANR Publication 8117; Managing mosquitoes in surface-flow contructed treatment wetlands). [https://anrcatalog.ucanr.edu/Details.aspx?itemNo=8117](https://urldefense.com/v3/__https:/anrcatalog.ucanr.edu/Details.aspx?itemNo=8117__;!!KGKeukY!2xiH3E-bhelpt-54KI2hnVHiqn4oAIYXZ4m1gCz20mCrWQa9HJuePFz0vAxFhQvco_uGd9GffaIOsmKEh4A$)
We agree this publication fits well with our discussion and has been added as a citation in lines 490 and 536.

o Line 518. Also mentioned above, I have yet to see a stormwater management structure / constructed treatment wetland that did not soon become a monoculture of cattails and /or bullrush. Although designs with fancy planting proposals look great on paper and are easy to sell, the maintenance required to keep the plant community as designed is beyond the scope and expertise of most municipalities. It's simply too labor intensive and specific in the majority of cases.

Text has been added to paragraph 473-495 to mention issues related to the difficulty in maintaining SCMs and preventing the spread of these plants.
